# High-injection effects in near-field thermophotovoltaic devices

**DOI:** 10.1038/s41598-017-15996-0

**Published:** 2017-11-20

**Authors:** Etienne Blandre, Pierre-Olivier Chapuis, Rodolphe Vaillon

**Affiliations:** 1 Univ Lyon, CNRS, INSA-Lyon, Université Claude Bernard Lyon 1, CETHIL UMR5008, F-69621 Villeurbanne, France; 2 Radiative Energy Transfer Lab, Department of Mechanical Engineering, University of Utah, Salt Lake City, UT 84112 USA

## Abstract

In near-field thermophotovoltaics, a substantial enhancement of the electrical power output is expected as a result of the larger photogeneration of electron-hole pairs due to the tunneling of evanescent modes from the thermal radiator to the photovoltaic cell. The common low-injection approximation, which considers that the local carrier density due to photogeneration is moderate in comparison to that due to doping, needs therefore to be assessed. By solving the full drift-diffusion equations, the existence of high-injection effects is studied in the case of a GaSb p-on-n junction cell and a radiator supporting surface polaritons. Depending on doping densities and surface recombination velocity, results reveal that high-injection phenomena can already take place in the far field and become very significant in the near field. Impacts of high injection on maximum electrical power, short-circuit current, open-circuit voltage, recombination rates, and variations of the difference between quasi-Fermi levels are analyzed in detail. By showing that an optimum acceptor doping density can be estimated, this work suggests that a detailed and accurate modeling of the electrical transport is also key for the design of near-field thermophotovoltaic devices.

## Introduction

The main advantage of the near-field thermophotovoltaic (NF-TPV) devices is the enhancement by orders of magnitude of the rate of photon absorption in the photovoltaic (PV) cell^1–3^. Indeed, when the vacuum gap distance between a thermal radiator and a cooler body is less than the characteristic wavelength of thermal radiation, tunneling of the evanescent modes gives rise to a near-field enhancement of thermal radiation transfer^4–6^. If the radiator supports surface polariton modes, radiation transfer takes place in a narrow spectral range in the near field^6^, with enhanced radiation transfer and in turn greater electrical power output in NF-TPV devices^7,8^. It is thus interesting to design a thermal radiator, which can be described by a Drude permittivity, so as to have its spectral resonance matching photon energies close to the bandgap of the cell^9^. Hence a significant amount of research focused on the spectral optimization of the coupling between the radiator and the PV cell^9–15^. These studies mostly included the calculation of the photocurrent and sometimes a radiative recombination current (i.e. a cell operating at the radiative limit) and assumed a fixed cell temperature. Certain analyses incorporated a simplified modeling of the non-radiative recombination of the electrical carriers. This was done using an analytical approximation of the photocurrent involving non-radiative recombination lifetimes^2,8,16^, or applying a detailed-balance analysis with non-radiative recombination^17,18^, or solving the minority carrier diffusion equation in the low-injection assumption^19–27^. Some articles of the latter category also dealt with the thermal behavior of the PV cell^21,24,26,27^. A couple of alternative near-field photoelectric converter devices not based on conventional layered p-n junction cells were also explored^28–30^.

In the aforementioned articles that consider an electrical transport modelling, application of the low-injection approximation, which states that the density of injected free carriers is moderate in comparison to that provided by the dopants^31^, was never challenged. Surprisingly, even though the near-field enhancement of photon absorption and electron-hole pair (EHP) injection is the backbone of NF-TPV devices, high-injection levels were not considered likely events. This may appear as an excessive assumption since radiation fluxes locally absorbed by the PV cell in a NF-TPV device largely exceed the one-Sun illumination level (1 kW·m^−2^) and may come close (e.g. up to 10 MW·m^−2^ in the extreme near field in ref.^19^) to the maximum illumination that solar concentrated PV cells allow (~46165 Suns, i.e. ~46.2 MW·m^−2^ ^32^). One possible reason is that high doping densities were assumed, thus making high-injection conditions rather unlikely. For example, following an analysis that applies to photodetectors^33^, Park *et al*.^19^ selected 10^19^ cm^−3^ and 10^17^ cm^−3^ for the acceptor (*N*_*a*_) and donor (*N*_*d*_) doping densities of the 0.4 μm thick p- and 10 μm thick n- layers respectively, of a In_0.18_Ga_0.82_Sb cell. Subsequent articles^21,22,24,26,27^ used the exact same doping densities (and the same thickness of the p- and n- layers) without questioning. Knowing that doping levels impact on the built-in voltage, the depletion layer thickness, the equilibrium carrier concentrations, carrier mobility, Auger and impurity [Shockley-Read-Hall (SRH)] recombination lifetimes, it follows that searching for the doping densities that maximize the electrical power output is a key question for the design of NF-TPV devices. For answering it, a specific electrical transport modelling is required since the low-injection approximation may become questionable when decreasing the doping densities.

Hence the main objectives of the present article are (i) to assess the existence of high-injection effects in near-field thermophotovoltaics and (ii) to analyze if optimal doping concentrations can be found. To achieve this, two electrical transport models are used: the common one which is valid only when the injection of electrical carriers is low – the Minority Carrier Separation (MCS) model –, the other one which is valid for any injection level of electrical carriers – the Full Drift-Diffusion (FDD) model– (see Methods). In this manuscript, the analysis is concentrated on the impact of varying the acceptor doping density on the electrical output power in the case of a GaSb p-on-n junction cell.

## Results

### Injection of electron-hole pairs in NF-TPV devices

**Figure 1:**
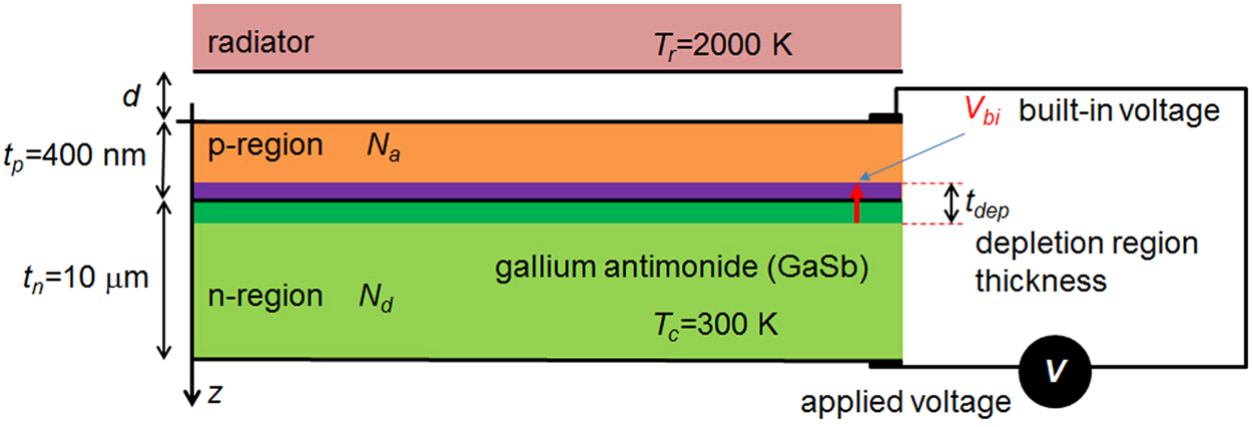
Main parameters of the NF-TPV device under consideration.

**Figure 2:**
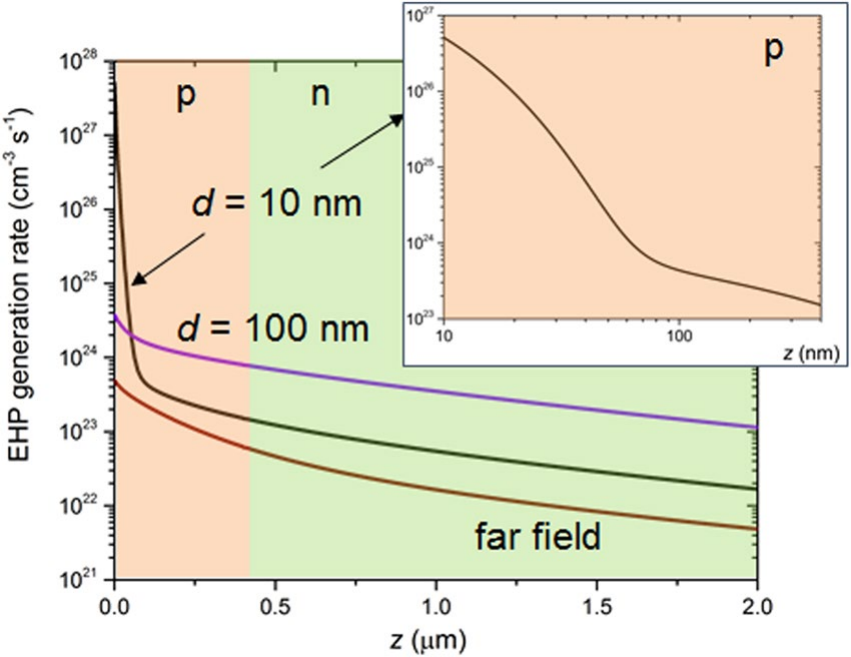
Electron-hole pair (EHP) generation rate as a function of depth in the cell, for three radiator-to-cell distances: in the far field, 100 nm and 10 nm.

A schematic of the NF-TPV device analyzed in the present article is given in Fig. 1. The cell is constituted of a single p-on-n junction made of gallium antimonide (GaSb) maintained at 300 K. For comparison purposes, most parameters of the configuration are chosen to be the same as those in a series of previous articles^19,21,22,24,26,27^, in particular the thickness of the p- and n- layers (*t*_*p*_ = 0.4 μm and *t*_*n*_ = 10 μm, respectively) and the temperature of the radiator (*T*_*r*_ = 2000 K). Validity of the low-injection approximation is assessed by varying the doping density of the p-region (*N*_*a*_, from 10^14^ cm^−3^ to 10^19^ cm^−3^) while the doping density of the n-region is fixed (*N*_*d*_ = 10^17^ cm^−3^). As in refs^18,24^, the radiator is made of a material supporting surface polariton modes, with the parameters of a Drude model for the complex permittivity, $\varepsilon (\omega )=1-{\omega }_{p}^{2}/({\omega }^{2}+i{\rm{\Gamma }}\omega )$, selected to maximize radiation transfer with energy larger than the bandgap of GaSb for a radiator-to-cell distance (*d*) of 10 nm (*ω*_*p*_ = 1.83 × 10^15^ rad/s and Γ = 2.10 × 10^13^ rad·s^−1^). The corresponding surface polariton resonance of an interface between such a Drude radiator and vacuum takes place at 0.85 eV, similarly to existing bulk materials (see Table 1 in ref.^15^). Compared to a broadband emitting radiator (e.g. tungsten as in refs^21,24^), such a radiator transfers thermal radiation to the cell less efficiently in the far field and in the near field down to a radiator-to-cell distance of 10 nm^24^, but generates huge near-field radiation absorption fluxes close to the front interface of the cell in the extreme near-field (*d* ≤ 10 nm). Accordingly, the profiles of local EHP generation rate (in cm^−3^·s^−1^), shown in Fig. 2 for the first two micrometers in the cell, are also changing drastically with the radiator-to-cell distance. The injection level is yet quite high in the far field (~10^23^ cm^−3^·s^−1^). It is increased by around one order of magnitude for a radiator-to-cell distance of 100 nm, and by four to five orders of magnitude close to the front surface of the cell (first 100 nanometers) for a radiator-to-cell distance of 10 nm.

**Table 1:** Cartesian one-dimensional steady-state equations ruling charge transport in the PV cell.

*n* current	${J}_{n}(z)=e\,n(z){\mu }_{n}E(z)+e\,{D}_{n}\frac{dn}{dz}\quad \quad \quad \quad \quad \quad \quad \quad \quad \quad \quad \quad \quad \quad \quad \quad \quad \quad \quad \quad \quad \quad \quad \quad \quad \,\,\,\,\,\,\,(1)$
*p* current	${J}_{p}(z)=e\,p(z)\,{\mu }_{p}\,E(z)-e\,{D}_{p}\,\frac{dp}{dz}\quad \quad \quad \quad \quad \quad \quad \quad \quad \quad \quad \quad \quad \quad \quad \quad \quad \quad \quad \quad \quad \quad \quad \quad \quad \,\,\,(2)$
Poisson’s equation	$\frac{dE}{dz}=-\frac{{d}^{2}V}{d{z}^{2}}=-\frac{e}{\varepsilon }[n(z)-p(z)+{N}_{a}(z)-{N}_{d}(z)]\quad \quad \quad \quad \quad \quad \quad \quad \quad \quad \quad \quad \quad \quad \quad \quad \quad \quad \,\,\,\,(3)$
continuity eq. for *n*	$-\frac{1}{e}\frac{d{J}_{n}(z)}{dz}=G(z)-R(z)\quad \quad \quad \quad \quad \quad \quad \quad \quad \quad \quad \quad \quad \quad \quad \quad \quad \quad \quad \quad \quad \quad \quad \quad \quad \quad \quad \quad \quad \,\,(4)$
continuity eq. for *p*	$\frac{1}{e}\frac{d{J}_{p}(z)}{dz}=G(z)-R(z)\quad \quad \quad \quad \quad \quad \quad \quad \quad \quad \quad \quad \quad \quad \quad \quad \quad \quad \quad \quad \quad \quad \quad \quad \quad \quad \quad \quad \quad \,\,\,\,\,(5)$
photo-generation rate	$G(z)={\int }_{0}^{\infty }{\kappa }_{\omega }^{IB}\frac{{q}_{\omega }^{inc}(z)}{\hslash \omega }d\omega \quad \quad \quad \quad \quad \quad \quad \quad \quad \quad \quad \quad \quad \quad \quad \quad \quad \quad \quad \quad \quad \quad \quad \quad \,\,\,\,\,\,\quad \quad \quad \quad (6)$
recombination rate	$R(z)={R}_{rad}\,(z)+{R}_{Auger}\,(z)+{R}_{SRH}\,(z)\quad \quad \quad \quad \quad \quad \quad \quad \quad \quad \quad \quad \quad \quad \quad \quad \quad \quad \quad \quad \quad \quad \,\,\,\,(7)$
radiative recombination rate	${R}_{{\rm{rad}}}\,(z)=B(n(z)p(z)-{n}_{i}^{2})\quad \quad \quad \quad \quad \quad \quad \quad \quad \quad \quad \quad \quad \quad \quad \quad \quad \quad \quad \quad \quad \quad \quad \quad \quad \quad \quad \,\,(8)$
Auger recombination rate	${R}_{{\rm{Auger}}}\,(z)={C}_{n}n(z)(n(z)p(z)-{n}_{i}^{2})+{C}_{p}p(z)(n(z)p(z)-{n}_{i}^{2})\quad \quad \quad \quad \quad \quad \quad \quad \quad \quad \quad \quad \quad \,\,\,\,(9)$
SRH recombination rate	${R}_{{\rm{SRH}}}\,(z)=\frac{n(z)p(z)-{n}_{i}^{2}}{{\tau }_{p}^{{\rm{SRH}}}(n(z)+{n}_{i}\,\exp [({E}_{t}-{E}_{i})/{k}_{b}T])+{\tau }_{n}^{{\rm{SRH}}}(p(z)+{n}_{i}\,\exp [({E}_{t}-{E}_{i})/{k}_{b}T])}\quad \quad \quad \quad \quad \quad \quad \quad \,\,\,\,(10)$
surface recombination	${R}_{{\rm{surf}}}\,({z}_{s})=\frac{{S}_{n}{S}_{p}(n({z}_{s})p({z}_{s})-{n}_{i}^{2})}{{S}_{n}(n({z}_{s})+{n}_{i})+{S}_{p}(p({z}_{s})+{n}_{i})}\quad \quad \quad \quad \quad \quad \quad \quad \quad \quad \quad \quad \quad \quad \quad \quad \quad \quad \quad \quad \quad \,\,\,(11)$

It is known that the local product of the electron (*n*) and hole (*p*) carrier densities should be such that $np\le {n}_{i}^{2}\exp (eV/kT)$ in any injection condition^31^, where *V* is the applied voltage, *e* the elementary charge and *n*_*i*_ the intrinsic carrier concentration. With an intrinsic carrier concentration for GaSb at 300 K of the order of 10^12^ cm^−3^ ^34^, and taking the bandgap of GaSb (0.726 eV at 300 K) as the maximum reachable *eV*, the product *np* cannot be in any case larger than approximately 1.6 10^36^ cm^−6^. Of course, the high injection rate is counterbalanced by a recombination rate, which grows as the product *np* grows for radiative, SRH and surface recombinations, and as *n* ^2^ *p* (and *np* ^2^) grows for Auger recombination (see Methods). In the case of NF-TPV devices, in particular those involving surface polariton modes with very small penetration depths of radiation within the cell^19^, surface recombination is also playing a major role^24^. For GaSb, the surface recombination velocity is reported to vary over four orders of magnitude from 1 to 2 10^4^ m·s^−1^ ^34,35^. Thus amplifying the EHP injection level, changing the doping concentration of the p-region, and varying the surface recombination velocity, are expected to generate complex combined effects on the performances of the cell.

### High injection effects

**Figure 3:**
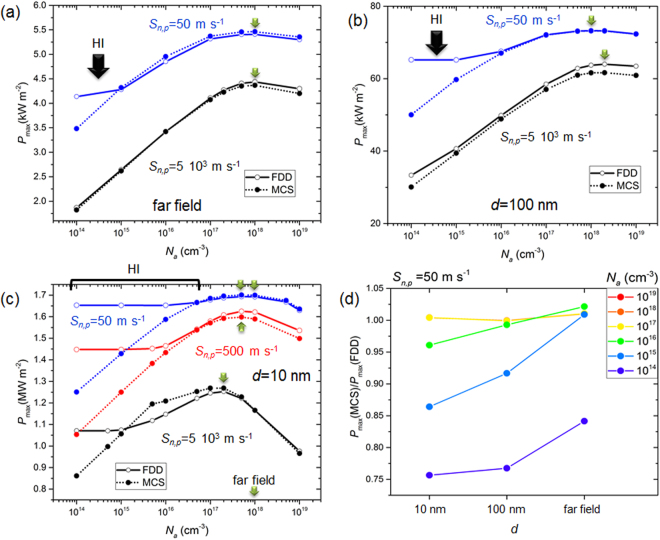
Electrical power density at the maximum power point (*P*_max_) as a function of the acceptor doping density (*N*_*a*_) for three radiator-to-cell distances: (**a**) in the far field; (**b**) *d* = 100 nm; (**c**) *d* = 10 nm. Simulations for a high (5 10^3^ m·s^−1^), an intermediate (500 m·s^−1^) and a low (50 m·s^−1^) surface recombination velocity. The optimum acceptor doping densities are indicated with a green arrow. (**d**) Ratio of electrical power density at the maximum power point (*P*_max_) calculated with the MCS model to that calculated with the FDD model, as a function of radiator-to-cell distance (*d*) for several acceptor doping densities (*N*_*a*_) and a low (50 m·s^−1^) surface recombination velocity.

Figure 3 shows the electrical power density at the maximum power point (*P*_max_) as a function the acceptor doping density (*N*_*a*_), for the three radiator-to-cell distances leading to the EHP injection rates depicted on Fig. 2. Simulations are reported for a high (*S*_*n,p*_ = 5 10^3^ m·s^−1^), an intermediate (*S*_*n,p*_ = 500 m·s^−1^), and a low (*S*_*n,p*_ = 50 m·s^−1^) surface recombination velocity. Results from both the model that assumes a low injection of electrical carriers (MCS) and the model valid for any injection level (FDD) are shown. In the far field, the magnitude of the maximum electrical power is of the order of kilowatts per square meter. With the additional contribution of the evanescent modes to thermal radiation transfer between the radiator and the cell, this order of magnitude becomes dozens of kilowatts and around one to two megawatts per square meter for radiator-to-cell distances of 100 and 10 nm, respectively.

In the far field (Fig. 3(a)), the relative difference between results from the MCS and FDD (the all-injection FDD model being the reference) is less than 2% except for one data point (*N*_*a*_ = 10^14^ cm^−3^ and *S*_*n,p*_ = 5 10^3^ m·s^−1^), where the low-injection approximation underestimates the maximum electrical power by 15%. This happens when the acceptor doping density and the surface recombination velocity are both low, thus suggesting that high-injection (HI) effects are taking place. One way to check this assertion is to calculate the ideality factor of the single diode junction model (see Methods). It is an indicator of the dominating recombination mechanisms and of the location where they mostly take place^31^. In the case of the low surface recombination velocity (5 10^3^ m·s^−1^), the ideality factor is equal to 1 at high acceptor doping concentrations (10^17^ to 10^19^ cm^−3^, see Fig. S1(a)). This is common in low-injection conditions^31^. For lower acceptor doping densities and a high surface recombination velocity, the ideality factor deviates from unity and reaches 1.8 at 10^14^ cm^−3^. This reveals that recombination is no more limited by a single type of carriers, meaning high-injection conditions are established^31^ (ultimately the ideality factor tends towards 2). An undisputable proof is given by looking at the variations with depth in the cell of electron and hole densities calculated at the maximum power point (*V* = *V*_max_) with the all-injection FDD model. For an acceptor doping density of 10^15^ cm^−3^, densities of both carriers are comparable in the p-region (see Supplementary Figure S1(b) and Supplementary Video), therefore making the definition of minority carriers inappropriate.

Figure 3(b) and (c) exhibit differences between the MCS and FDD models that are taking place over a larger range of acceptor doping density than in the far field, depending on both the radiator-to-cell distance and the surface recombination velocity. At large acceptor doping densities, the differences between the models are smaller than 10%. When high injection is taking place, i.e. at the lowest acceptor doping densities, the difference is much larger. For example, in the extreme near field (*d* = 10 nm), the low-injection approximation may lead to underestimating the maximum electrical power by up to 25% (see also Fig. 3(d)). Since the generation rates of EHP are one to several orders of magnitude larger than in the far field, it is not surprising that high-injection effects are taking place at larger acceptor densities in the near field. Because EHP are injected close to the front surface of the cell, a smaller surface recombination velocity means that more of them do not recombine immediately and can participate to the charge transport. Hence high injection can take place at even larger acceptor densities in the near field. In Fig. 3(d) a plot as a function of radiator-to-cell distance of the ratio of electrical power density at the maximum power point calculated with the MCS model to that calculated with the FDD model, provides another way to observe this effect and its scale.

**Figure 4:**
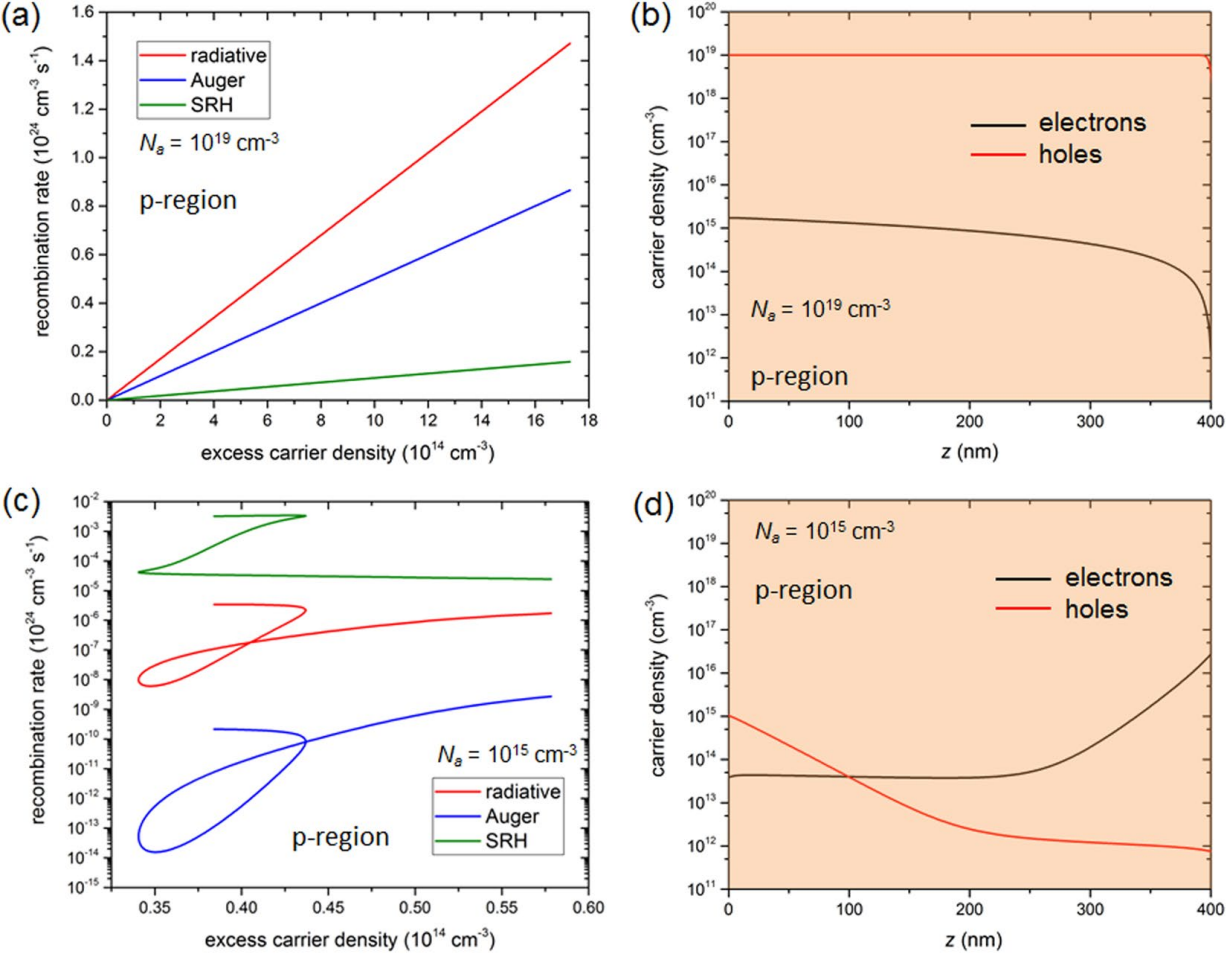
(**a**,**c**) Bulk recombination rates as a function of the excess carrier (electron) density in the p-region. (**b**,**d**) Electron and hole densities a function of depth in the cell in the p-region. Simulations made with the FDD model at *V* = 0 for *d* = 10 nm, a high (5 10^3^ m·s^−1^) surface recombination velocity, a low-injection (*N*_*a*_ = 10^19^ cm^−3^) and a high-injection (*N*_*a*_ = 10^15^ cm^−3^) condition.

The main explanation for the failure of the MCS model is that it predicts a drop of the open-circuit voltage following that of the built-in-voltage, while the FDD model does not (see section 1.2. of the Supplementary Information). In high-injection conditions, an electric field builds in the p-region thus allowing a larger number of photogenerated electrons to be collected without being recombined, even at voltages larger than the built-in-voltage. Since the FDD model calculates the spatial distributions of the bulk recombination rates and carrier densities, it is possible to compute the local recombination rates as a function of the excess carrier density of electrons in the p-region, in the extreme near field, for a low-injection (Fig. 4(a)) and a high-injection (Fig. 4(c)) regime. One of the assumptions of the MCS model (see Methods) is confirmed by simulations made with the FDD model: the recombination rate depends linearly on the excess minority carrier density. For each recombination process, the slope is found to be exactly the inverse of the corresponding electron lifetime. However, in high-injection conditions, each recombination rate depends non-linearly on electron carrier density, and obviously also on hole carrier density, which are not constant across the p-region (Fig. 4(d)). It is worth noticing that both the levels and the hierarchy of the bulk recombination processes are strongly affected by the injection regime. As no photon recycling factor is applied in these simulations, radiative recombination rates are quite high. For the low acceptor doping density, the hole density is as low as the electron density across the p-region and much smaller than for a high acceptor doping density (Fig. 4(b) and (d)). Hence mostly Auger and then radiative recombination rates are much lower in the case with high-injection effects.

**Figure 5:**
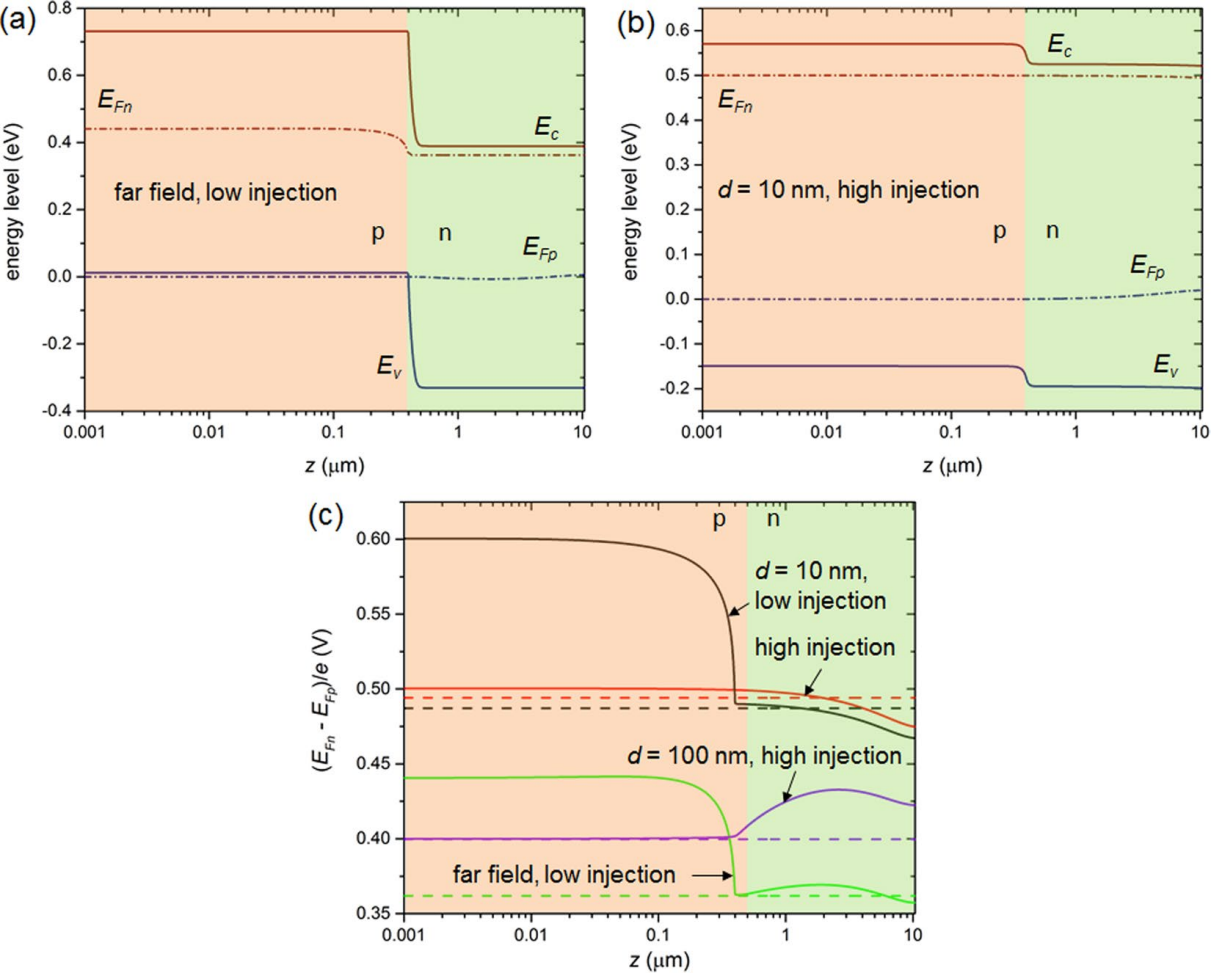
Band diagram in (**a**) a far-field configuration where low injection holds (*N*_*a*_ = 10^19^ cm^−3^, *S*_*n,p*_ = 5 10^3^ m·s^−1^), and (**b**) a near-field configuration where high injection is observed (*d* = 10 nm, *N*_*a*_ = 10^15^ cm^−3^ and *S*_*n,p*_ = 50 m·s^−1^). (**c**) Difference between the quasi-Fermi levels as a function of depth within the cell in two low-injection (*N*_*a*_ = 10^19^ cm^−3^, *S*_*n,p*_ = 5 10^3^ m·s^−1^; in green for the far field and in black for *d* = 10 nm) and two high-injection (*N*_*a*_ = 10^15^ cm^−3^ and *S*_*n,p*_ = 50 m·s^−1^; in purple for *d* = 100 nm and in red for *d* = 10 nm) cases. Results obtained using the FDD model at *V* = *V*_max_ (dashed lines in (**c**)).

The all-injection FDD model also calculates the quasi-Fermi levels for electrons and holes (*E*_*Fn*_ and *E*_*Fp*_) across the cell. It is known that in idealized conditions, the difference of these levels should be equal to *eV* in the depletion region^31,36^, where *V* is the applied voltage and *e* the elementary charge. Figure 5(a) and (b) display the band diagram calculated using the FDD model at the operating voltage (*V*_max_), for a low-injection and a high-injection configuration, respectively. The potential barrier at the junction is large in the low-injection case as expected, and weak but nonzero in the high-injection case. Figure 5(c) depicts the corresponding differences (*E*_*Fn*_ − *E*_*Fp*_)/*e*, together with those for other low- or high- injection cases. It is worth noticing that the difference in quasi-Fermi levels may deviate significantly from the applied voltage, in particular near the front of the cell in the two low-injection conditions. In high-injection conditions, the divergence from the applied voltage is weak in the p-region, but may become significant in the n-region.

### Optimum acceptor doping density

The previous analysis assessing high-injection effects in NF-TPV devices reveals that the acceptor doping density can be tuned to maximize the electrical power output. In the far field, there is an optimum value of the acceptor doping density (10^18^ cm^−3^) which does not depend on the surface recombination velocity. For acceptor doping densities decreasing down to 10^14^ cm^−3^, the maximum electrical power decreases linearly. In the frame of the low-injection approximation (MCS model), this optimum is explained by the counteracting effects taking place in the cell (details given in Sect. 2.1. of the Supplementary Information). For a fixed donor doping density in the n-region, lowering the acceptor doping density in the p-region leads to: (i) a larger depletion region moving toward the p-region and eventually fully covering it; (ii) a larger mobility of electrons which are the minority carriers in the p-region; (iii) a weaker built-in voltage. The first two phenomena make the short circuit current density increase until reaching saturation (when the p-region is fully depleted). The third one means that the potential barrier of the junction becomes less effective, thus generating a drop of the open-circuit voltage. Plots of current-voltage and power-voltage characteristics as a function of acceptor doping density (Supplementary Figure S5) illustrate these effects and confirm the existence of a doping density maximizing the electrical power.

The optimum acceptor doping density for a radiator-to-cell distance of 100 nm is close to that in the far field (2 10^18^ cm^−3^ for *S*_*n,p*_ = 5 10^3^ m·s^−1^), and spans over a range [5 10^17^–2 10^18^] cm^−3^ when the surface recombination velocity is decreased to a lower value (*S*_*n,p*_ = 50 m·s^−1^). In the extreme near field (*d* = 10 nm), the spatial distribution of EHP generation rate is very different because of the surface polaritons. A huge amount of EHP is generated in the first hundred nanometers of the p-region of the cell, with a very sharp decrease of the generation rate over this depth (more than three orders of magnitude). Hence the curve of Fig. 3(c) for a high surface recombination velocity (5 10^3^ m·s^−1^) reveals an optimum acceptor doping level (2 10^17^ cm^−3^) smaller than those for the previous radiator-to-cell distances. An in-depth analysis (see section 2.2. in the Supplementary Information) provides explanations for the dependence of the optimum acceptor doping density on near-field radiation transfer when the surface recombination velocity is high. In summary, the different regimes of radiative heat transfer between the radiator and the cell govern the level and gradient in the cell of the EHP generation rate (Fig. 2). As a consequence, modifications caused by varying the acceptor doping density, e.g. the position of the quasi-neutral and depletion regions, have an impact on how many of these injected charges are collected. Indeed, for the high surface recombination velocity, the short-circuit current density is the most affected (Supplementary Figure S6(a)) by these changes.

The optimum acceptor doping density is also sensitive to the surface recombination velocity in the extreme near field case (*d* = 10 nm, Fig. 3(c)). Again the spatial distribution of the EHP generation rate matters. High surface recombination means that a lot of the charges generated close to the surface cannot contribute to the photocurrent. Hence a low acceptor doping, leading to a shift of the depletion region towards the surface, is beneficial, even though the open-circuit voltage drops. This is less the case when surface recombination decreases, explaining why the optimum doping density shifts towards larger values. Without the specific distribution of EHP generation rate caused by the surface polariton modes, these effects are much less important, explaining why the optimum doping density is not much sensitive to surface recombination at larger radiator-to-cell distances.

## Discussion

The results suggest that the simple electrical models of NF-TPV devices, such as the detailed balance approach (or alternatively the single junction diode equation), should be used with caution. Using or even proposing approximate formulations for the emission current (or diode saturation current) and the ideality factor, as in refs^37,38^, would be possible, but this would require detailed extensive analyses taking into account the specific near-field radiation absorption regime. Solving in details for the transport of electrical charges in the cell seems essential. Given the huge level of radiation absorption leading to peculiar EHP generation rates in NF-TPV devices, the present work has investigated on the validity of the low-injection approximation. It should be noted that differences larger than 10% between the FDD and MCS model results have been found only in cases where the acceptor doping density is moderate, and often smaller than the one maximizing electrical power output. It might be tempting at first to conclude that the MCS model can apply in any circumstances. However, it was shown that in high-injection conditions, the cell can operate very differently: an electric field can be built over the whole p-region, a potential barrier can be sustained despite low acceptor doping densities, recombination rates depend on both carriers, and the difference between the quasi-Fermi levels across the entire cell is altered. The assessment with an all-injection, i.e. the FDD, model seems unavoidable in this case.

The results have revealed that the difference between the quasi-Fermi levels across the cell may differ from the product of the elementary charge with the applied voltage. In photovoltaics, it is however routinely taken for granted that this equality applies to the whole cell (e.g. refs^39–41^), and in particular for calculating emission due to radiative recombination in NF-TPV devices^9,11–15,17,18^, by using the generalized Planck function^42^. It is worth mentioning also that no recycling factor has been inserted in the radiative recombination term in the present work. In practice, a recent article^18^ shows that such a factor should vary as a function of depth in the cell, in particular in the case of the same Drude radiator (for *d* = 10 nm, between 10^−2^ and ~10^3^). The last two remarks call for elaborating a refined model for radiative recombination. The all-injection model would clearly be required for analyzing in details such physics.

Similarly to the case of solar cells, the radiative behavior of the NF-TPV cell is the primary driver for the electrical performances of the devices. The initial idea is that as much as possible radiation energy from the radiator should contribute to generating EHP in the cell. Here, it has been shown that the link between the spatial distribution of the EHP generation rate in the cell and the main characteristics of the cell (doping densities, size of the doped layers and surface passivation) is also of outmost importance. This is better evidenced for NF-TPV because for conventional solar cells and even concentrated photovoltaics the generation of EHP does not vary as strongly. The existence of an optimum acceptor doping density, which depends on both the radiator-to-cell distance and the surface recombination velocity – and more generally on the spatial distribution of the EHP generation rate in the cell - has been highlighted by means of the use of a detailed and accurate electrical model. Thus the coupling of the radiative transfer model and of the electrical transport model is key for the design of NF-TPV devices.

One obvious extension of the present work would be to perform extensive simulations to determine all together optimum thicknesses of the p- and n- regions, and both the acceptor and donor doping densities, for various radiator materials and temperatures. Also, using lower bandgap semiconductors, such as indium antimonide (InSb, *E*_*g*_ = 0.17 eV at 300 K), would in principle allow converting more infrared photons. However, at high carrier densities these semiconductors become degenerate, meaning that substantial modifications of the MCS and FDD models would be required before analyzing high-injection effects, and performing an optimal design of the photovoltaic cell.

## Methods

### Radiative transfer modeling

Fluctuational electrodynamics^43^ and the S-matrix method for layered media^44^ are used to compute the spatial distribution of EHP generation rate in the cell. Calculations are made in the frequency interval [7.53 10^14^, 37.63 10^14^] rad·s^−1^ ([0.496, 2.5] eV). The dielectric function for frequencies larger than that of the bandgap of GaSb at 300 K (10.98 10^14^ rad·s^−1^, i.e. 0.723 eV) are given by the model from Adachi^45^. The dielectric function for the interactions of radiation with the lattice and free carriers, expressed as a Lorentz-Drude model with parameters from^46^, is assumed negligible compared to that for interband transitions.

### FDD and MCS models

The set of equations required to model the 1D Cartesian steady-state drift, diffusion, generation, recombination of electrons (*n*) and holes (*p*) within a photovoltaic cell under all-injection conditions, summarized in Table 1, are available from textbooks (e.g. refs^31,47,48^). Most variables are common in semiconductor device modeling and thus detailed explanations are not provided. The parameter specific to NF-TPV device modeling is the local total generation rate of EHP given by eq. (6), where *z* is the depth in the cell, ${\kappa }_{\omega }^{IB}$ is the interband absorption coefficient, and ${q}_{\omega }^{inc}(z)$ is the local spectral incident flux, calculated using the radiative transfer model described previously. Physical parameters such as the intrinsic carrier concentration (*n*_*i*_), mobilities (*μ*) and diffusion coefficients (*D*) are calculated at the temperature of the cell (300 K). The dependence of electron and hole mobility on doping is taken into account by means of the Caughey-Thomas model^34^. The radiative recombination coefficient *B* is taken from^34^ without considering any photon recycling factor. Values given in ref.^35^ are used for the Auger recombination coefficients *C*_*n*_ and *C*_*p*_. The SRH lifetimes required in eq. (10) are computed using the expression found in ref.^33^. It is common to assume that the energy of traps is equal to that of the intrinsic energy level (i.e. *E*_*t*_ = *E*_*i*_). In eq. (11), surface recombination involves the surface recombination velocity of electrons and holes, which typically varies between 1 and 2 10^4^ m·s^−1^ for GaSb^34,35^. Boundary conditions are established for Poisson’s equation by assuming space charge neutrality at the edges of the cell. For continuity equations, boundary conditions state that two quasi-Fermi levels are set by the potential at the edges of the cell (zero for that of holes at the top of the p-region, -*V* for that of electrons at the bottom of the n-region), and two carrier currents are counterbalanced by surface recombination at the edges of the cell (electron current at the top of the p-region, hole current at the bottom of the n-region).

The drift-diffusion equations (1–5) together with their boundary conditions are particularly difficult to solve for the unknowns (*n*, *p*, *E*) because of strong coupling and non-linearities. Thus for numerical stability purposes, a normalized electrostatic potential and the so-called Slotboom variables^49,50^ are introduced. Gummel’s iterative method^51^ is used to solve the modified drift-diffusion equations. These equations and their numerical solution are referred to as the FDD model.

Under the low-injection approximation, the previous equations are significantly simplified. By assuming that the density of free carriers is much smaller than doping densities everywhere, *n* and *p* can be removed in Poisson’s equation (3). There is an electric field only in the depletion region at the junction between the n- and p-doped zones. In the n-doped (resp. p-doped) region, photogenerated holes (resp. electrons) are minority carriers that move only by diffusion. Thus current (1,2) and continuity equations (4,5) are greatly condensed. As the low-injection assumption is routinely used in photovoltaics, and applied to NF-TPV devices in several articles^19–27^, the simplified equations and their numerical solution, referred to as the MCS model, are not presented in details. It is worth pointing out that recombination rates are expressed as the ratio of excess carrier density to the total lifetime derived using a Mathiessen’s rule. The lifetimes for electrons and holes of each bulk recombination mechanism (radiative, Auger, SRH) are calculated to be consistent with the parameters of the recombination rates in the FDD model. The depletion region width (*t*_*dep*_) and its distribution within the n- and p- regions, which depend on the acceptor and donor doping concentrations, the built-in (*V*_*bi*_) and applied (*V*) voltages, are properly calculated (see e.g. ref.^31^). The p-region may become fully depleted for low acceptor doping densities (e.g. when *N*_*a*_ = 10^15^ cm^−3^, see Supplementary Figure S4(a)). In this case, since the width of the depletion region in the p-region cannot exceed that of the p-region itself, the width of the depleted zone in the n-region is altered to ensure electroneutrality of the whole depletion region. The photocurrent and the dark current in the depleted p-region are thus calculated by solving a drift-diffusion equation involving a uniform electric field *E*, equal to (*V*_*bi*_-*V*)/*t*_*dep*_.

It is worth mentioning that in the implementation of the MCS model, the total photocurrent is calculated as the sum of photocurrents calculated by solving the diffusion equation for the minority carriers in the non-depleted parts of p- and n-regions, using EHP generation rates determined for a selected number of discrete intervals covering the spectral range of interest. The dark current comes from the solution of the diffusion equation for the majority carriers, calculated for a discrete number of applied voltages. By applying the superposition principle, the current density-voltage characteristics results from the difference of the total photocurrent and the dark current. With the FDD model, even though the radiative transfer problem is solved for the same number of discrete spectral intervals, the total EHP generation rate is the appropriate input in the drift-diffusion equations. These equations are solved for electrons and holes in the cell without considering any depletion region, for a discrete number of applied voltages, starting from zero and until reaching the open-voltage, i.e. when generation is balanced by recombination in the entire cell.

For both the FDD and the MCS models, the current density-voltage characteristics and in turn the maximum power output are the final output of the simulations. The parameters of the single diode model, including the ideality factor, which fit the resulting current density-voltage characteristic, are determined using the five-point method described in ref.^52^.

## Electronic supplementary material

Supplementary Information

Supplementary Video

## References

[1] Pan JL (2000). Very large radiative transfer over small distances from a black body for thermophotovoltaic applications. IEEE Trans. Elec. Dev..

[2] Whale MD, Cravalho EG (2002). Modeling and performance of microscale thermophotovoltaic energy conversion devices. IEEE Trans. Energy Convers..

[3] Greffet J-J (2016). Near-field radiative transfer in the energy-conversion schemes. Section 16 in Boriskina, S.V. *et al*. Roadmap on optical energy conversion. J. Opt..

[4] Polder D, Van Hove M (1971). Theory of radiative transfer between closely spaced bodies. Phys. Rev. B.

[5] Loomis JJ, Maris HJ (1994). Theory of heat transfer by evanescent electromagnetic waves. Phys. Rev. B.

[6] Mulet J-P (2002). Enhanced radiative heat transfer at nanometric distances. Microscale Thermophys. Eng..

[7] Narayanaswamy A, Chen G (2003). Surface modes for near field thermophotovoltaics. Appl. Phys. Lett..

[8] Laroche M (2006). Near-field thermophotovoltaic energy conversion. J. App. Phys..

[9] Ilic O (2012). Overcoming the blackbody limit in plasmonic and graphene near-field thermophotovoltaic systems. Opt. Express.

[10] Basu S (2007). Microscale radiation in thermophotovoltaic devices - A review. Int. J. Energy Research.

[11] Zhai X (2010). Performance analysis of thermophotovoltaic system with an equivalent cut-off blackbody emitter. J. App. Phys..

[12] Messina R, Ben-Abdallah P (2013). Graphene-based photovoltaic cells for near-field thermal energy conversion. Scientific Reports.

[13] Svetovoy VB, Palasantzas G (2014). Graphene-on-silicon near-field thermophotovoltaic cell. Phys. Rev. Applied.

[14] Karalis A, Joannopoulos JD (2015). Temporal coupled-mode theory model for resonant near-field thermophotovoltaics. Appl. Phys. Lett..

[15] Karalis A, Joannopoulos JD (2016). ‘Squeezing’ near-field thermal emission for ultra-efficient high-power thermophotovoltaic conversion. Scientific Reports.

[16] Tong JK (2015). Thin-film ‘thermal well’ emitters and absorbers for high-efficiency thermophotovoltaics. Scientific Reports.

[17] Chen K (2015). Suppressing sub-bandgap phonon-polariton heat transfer in near-field thermophotovoltaic devices for waste heat recovery. Appl. Phys. Lett..

[18] DeSutter, J. *et al*. External luminescence and photon recycling in near-field thermophotovoltaics. *Phys. Rev. Applied* **8**, 014030 (2017).

[19] Park K (2008). Performance analysis of near-field thermophotovoltaic devices considering absorption distribution. J. Quant. Spectrosc. Radiat. Transfer.

[20] Basu S (2009). Review of near-field thermal radiation and its application to energy conversion. Int. J. Energy Research.

[21] Francoeur M (2011). Thermal impacts on the performance of nanoscale-gap thermophotovoltaic power Generators. IEEE Trans. on Energy Conversion.

[22] Bright TL (2014). Performance of near-field thermophotovoltaic cells enhanced with a backside reflector. J. Heat Transfer.

[23] Lim M (2015). Graphene-assisted Si-InSb thermophotovoltaic system for low temperature applications. Opt. Express.

[24] Bernardi MP (2015). Impacts of propagating, frustrated and surface modes on radiative, electrical and thermal losses in nanoscale-gap thermophotovoltaic power generators. Scientific Reports.

[25] Jin S (2016). Hyperbolic metamaterial-based near-field thermophotovoltaic system for hundreds of nanometer vacuum gap. Opt. Express.

[26] Lau JZ-J (2016). Parametric investigation of nano-gap thermophotovoltaic energy conversion. J. Quant. Spectrosc. Radiat. Transfer.

[27] Elzouka M, Ndao S (2017). Towards a near-field concentrated solar thermophotovoltaic microsystem: Part I – Modeling. Solar Energy.

[28] Wu DM (2009). Quantum-coupled single-electron thermal to electric conversion scheme. J. App. Phys..

[29] Molesky S, Jacob S (2015). Ideal near-field thermophotovoltaic cells. Phys. Rev. B.

[30] St-Gelais R (2017). Hot carrier-based near-field thermophotovoltaic energy conversion. ACS Nano.

[31] Sze, S. M. & Ng, K. K. Physics of semiconductor devices. Third edition, John Wiley & Sons, Hoboken (2007).

[32] Nicolás RO, Durán JC (1984). Theoretical maximum concentration factors for solar concentrators. J. Opt. Soc. America A.

[33] González-Cuevas JA (2006). Modeling of the temperature-dependent spectral response of In_1−χ_Ga_χ_Sb infrared photodetectors. Opt. Engineering.

[34] Martín D, Algora C (2004). Temperature-dependent GaSb material parameters for reliable thermophotovoltaic cell modelling. Semicond. Sci. Technol..

[35] Stollwerk G (2000). Characterization and simulation of GaSb device-related properties. IEEE Trans. on Elec. Dev..

[36] Green, M. A. Third generation photovoltaics. Advanced solar energy conversion. Springer, Berlin (2003).

[37] Scheer, R. & Schock, H.-W. Chalcogenide photovoltaics. Physics, technologies and thin film devices. Wiley-VCH Verlag GmbH & Co, 368 p. (2011).

[38] Cuevas A (2014). The recombination parameter J_0_. Energy Procedia.

[39] Martí A (1997). Photon recycling and Shockley’s diode equation. J. Appl. Phys..

[40] Hirst LC, Ekins-Daukes NJ (2011). Fundamental losses in solar cells. Prog. Photovoltaics Res. Appl..

[41] Dupré (2015). Physics of the temperature coefficients of solar cells. Sol. Energy Mat. Sol. Cells.

[42] Würfel P (1982). The chemical potential of thermal radiation. J. Phys. C: Solid State Phys..

[43] Rytov, S. M. *et al*. Principles of Statistical Radiophysics 3: Elements of Random Fields. Springer New York (1989).

[44] Francoeur (2009). R. Solution of near-field thermal radiation in one-dimensional layered media using dyadicGreen’s functions and the scattering matrix method. J. Quant. Spectrosc. Ra..

[45] Adachi S (1989). Optical dispersion relations for GaP, GaAs, GaSb, InP, InAs, InSb, A_*l*_x G_*a1−*x_ As, and I_*n1−*x_ G_*a*_x A_*s*_y _*P1−*y_. J. Appl. Phys..

[46] Patrini M (1997). Optical functions of bulk and epitaxial GaSb from 0.0025 to 6 eV. Solid State Commun..

[47] Green, M. A. Solar cells: operating principles, technology and system applications. University of New South Wales (1986).

[48] Streetman, B. & Banerjee, S. Solid State Electronic Devices. 6^th^ edition, Pearson Prentice Hall (2006).

[49] Slotboom JW, Philips NV (1973). Computer-aided two-dimensional analysis of bipolar transistors. IEEE. T. Electron. Dev..

[50] Vasileska, D. *et al*. Computational Electronics: Semiclassical and Quantum Device Modeling and Simulation, CRC Press (2016).

[51] Gummel HK (1964). A self-consistent iterative scheme for one-dimensional steady state transistor calculations. IEEE Trans. Electron Dev..

[52] Chan DSH, Phang JCH (1987). Analytical methods for the extraction of solar-cell single- and double-diode model parameters from I-V characteristics. IEEE Trans. Electron Dev..

