# Supplementary material for: High-injection effects in near-field thermophotovoltaic devices
Source: Sci Rep. 2017 Nov 20;7:15860. doi: 10.1038/s41598-017-15996-0 (PMC5696483; doi:10.1038/s41598-017-15996-0)
Supplement: Supplementary file 1 — Supplementary Information [file 41598_2017_15996_MOESM1_ESM.pdf]

High-injection effects in near-field thermophotovoltaic devices

Supplementary Information

Etienne Blandre,[1] Pierre-Olivier Chapuis,[1] and Rodolphe Vaillon,[1,2,a)]

[1]Univ Lyon, CNRS, INSA-Lyon, Université Claude Bernard Lyon 1, CETHIL UMR5008, F-69621, Villeurbanne, France

[2]Radiative Energy Transfer Lab, Department of Mechanical Engineering, University of Utah, Salt Lake City, UT 84112, USA

1. High injection: illustration and impacts

1.1. High-injection conditions illustrated in the far field

The ideality factor (*n*) of a photovoltaic cell is an indicator of the dominant recombination processes and of where they take place (in the quasi-neutral or space charge regions). It also indicates if recombination involves one type of injected carriers (low injection, *n*=1) or both types (high injection, *n*=2) [R1]. Supplementary Figure S1 (a) clearly indicates the transition from low-injection conditions (high acceptor doping densities) to high-injection conditions (lower acceptor doping densities), in the configuration where the radiator-to-cell distance corresponds to the far-field regime of thermal radiation, and for a low surface recombination velocity (50 m·s^{-1}). Variations of the electron and hole densities with depth in the cell, calculated with the FDD model for N_a = 10^{15} cm^{-3}, N_d = 10^{17} cm^{-3} and $S_{n,p}$ = 50 m·s^{-1} and V=$V_{\max}$, confirm that high-injection conditions are met in the p-region (Supplementary Figure S1 (b)). Both carrier densities are comparable, thus making inappropriate the separation of majority carriers from minority carriers. The Supplementary Video provides an animation with figures similar to Supplementary Figure S1 (b), where the acceptor doping density varies from 10^{14} to 10^{19} cm^{-3}.

a) Correspondence and requests for materials should be addressed to R.V. (rodolphe.vaillon@insa-lyon.fr or rodolphe.vaillon@utah.edu).

The acceptor doping densities for which it is possible to define majority and minority carriers in the p-region are clearly visible.

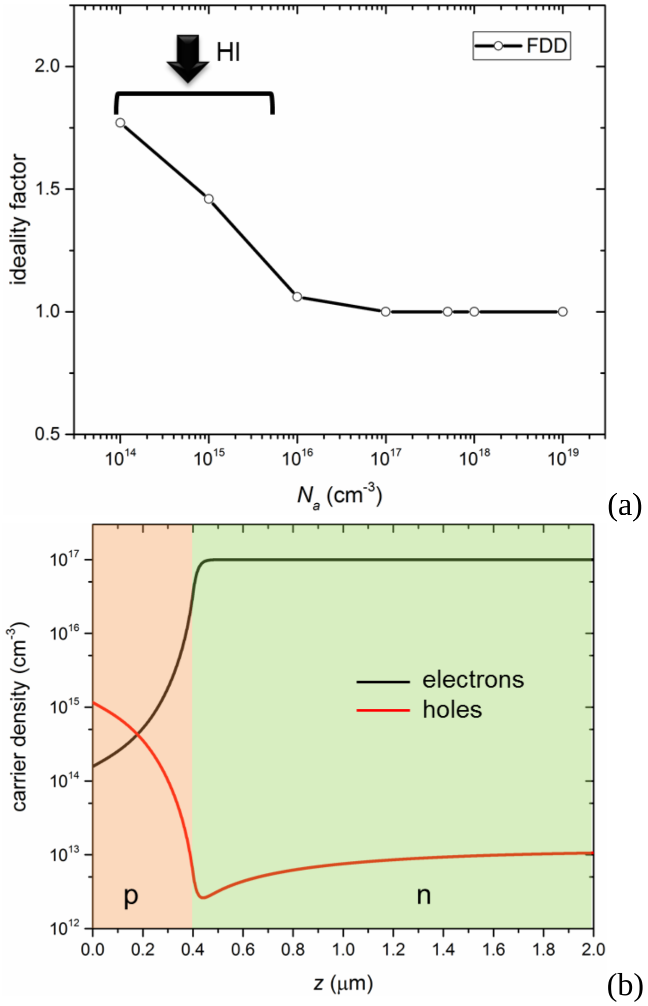

Supplementary Figure S1. (a) Single diode model ideality factor as a function of acceptor doping density (N_a). (b) Electron and hole densities as a function of depth in the cell for N_a = 10^{15} cm^{-3} and V=V_{max}. Calculations made using the FDD model for a far-field radiator-to-cell distance, N_d = 10^{17} cm^{-3} and $S_{n,p}$ = 50 m·s^{-1}.

1.2. The open-circuit voltage drop is shifted in high-injection conditions

The MCS model assumes the existence of quasi-neutral regions where the doping densities are responsible for providing majority carriers (holes in the p-region, electrons in the n-region), the opposite sign carrier being a minority one. As a consequence, a voltage drop occurs only between the edges of the depletion layer where a quasi-constant electric field is built within it.

The potential barrier created at the junction - the built-in voltage - drops when doping densities decrease [R1]. When a forward bias is applied, this barrier is lowered. Diffusion of majority carriers across the junction increases, leading to more recombination. The final consequence is a drop of the open-circuit voltage, the voltage at which recombination balances generation in the cell. This drop predicted in the frame of the MCS model is clearly observed in Supplementary Figs. S2 (a) and (b), for the open-circuit voltage (V_{oc}) and in turn for the voltage at the maximum power point (V_{max}). However, the FDD model provides a different behavior of the open-circuit voltage and the voltage at the maximum power point when the acceptor doping density decreases. They both reach a lower limit in the acceptor doping density range where high-injection effects are taking place (below 10^{16} cm^{-3} in Supplementary Fig. S2 (a) and below 10^{17} cm^{-3} in Supplementary Fig. S2 (b)).

The FFD model is a powerful simulation tool as it allows the determination of the spatial distribution in the cell of the potential, the electric field, and electron and hole densities. Supplementary Figs. S3 (a) and (b) depict the variations of the potential within the first two micrometers of the cell and the corresponding electric field, in a low-injection configuration (in the far field, for a moderate acceptor doping concentration N_a = 10^{17} cm^{-3} and a high surface recombination velocity $S_{n,p}$ = 5 10^3 $m\cdot s^{-1}$). When no forward bias (V = 0) is applied, the potential jumps at the junction and reaches the built-in voltage (V_{bi} = 0.586 V). The electric field is localized at the junction, in the depletion layer. When the bias is larger, as expected the potential barrier is lower and the electric field remains localized close to the junction. Results are quite different in a high-injection configuration (d = 10 nm, for a small acceptor doping concentration N_a = 10^{15} cm^{-3} and a low surface recombination velocity $S_{n,p}$=50 $m\cdot s^{-1}$), as illustrated in Supplementary Figs. S3 (c) and (d). When no forward bias (V = 0) is applied, the potential still reaches the built-in voltage (V_{bi} = 0.467 V), but by increasing continuously through the whole p-region. A large electric field is established in the whole p-region. At $V = V_{\mathrm{max}}$, equal to 0.494 V according to the FDD model, the MCS model is not applicable, because a voltage larger than the built-in voltage cannot be implemented in the model. However, when there is a huge EHP generation rate and low acceptor doping concentrations, density of injected electrons is comparable to that of holes in the p-region, and a gradient of both charges is established over the whole region. As a result, an electric field is sustained and a potential barrier still holds. A sufficiently large amount of electrons in the p-region, not recombined at the surface (hence the

essential role of lowering the surface recombination velocity $S_{n,p}$), are swept towards the n-region by means of that field. The overall generation rate remains larger than the overall recombination rate, although recombination is also highly promoted by a lower potential barrier that allows the diffusion of more electrons and holes across the junction (and thus a larger diode current opposite to the photocurrent).

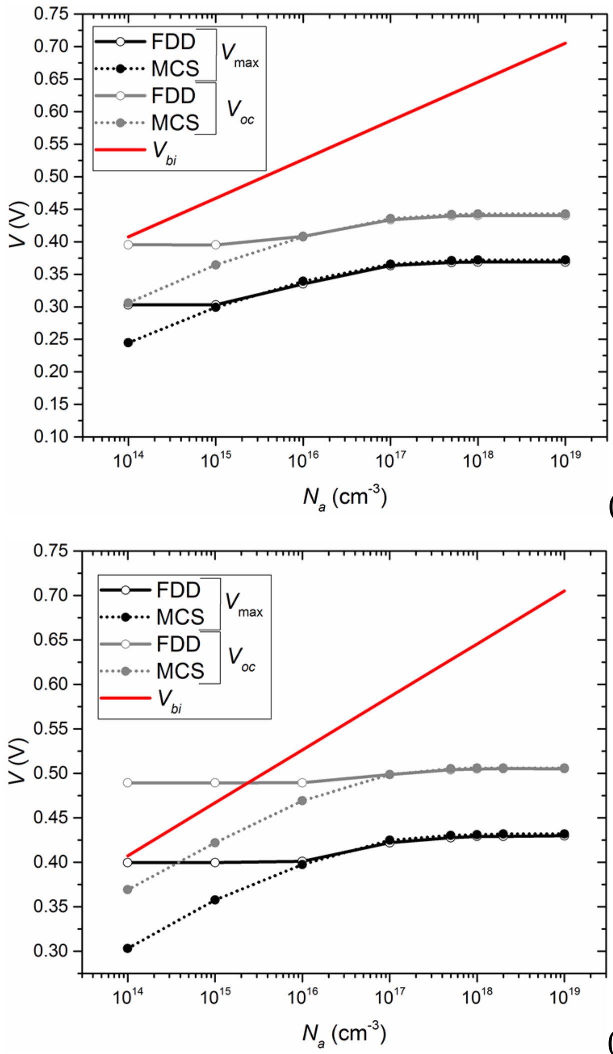

(a)

(b)

Supplementary Figure S2. Open-circuit voltage and voltage at maximum power point as a function of the acceptor doping density (N_a) for radiator-to-cell distances: (a) in the far field; (b) *d*=100 nm. Simulations made using the FDD model for a low (50 m·s^{-1}) surface recombination velocity.

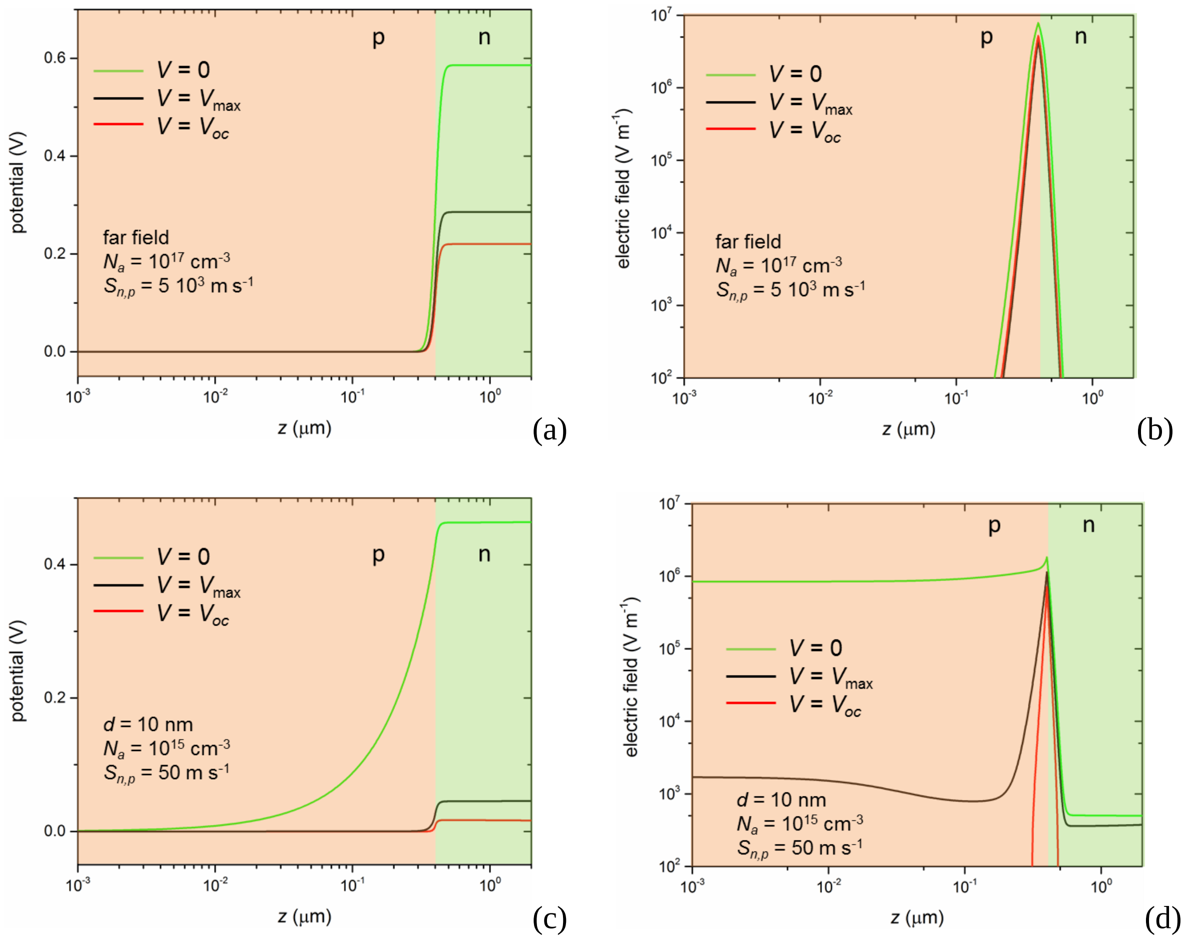

Supplementary Figure S3. Potential and electric field across the first two micrometers of the cell. (a, b) in low-injection conditions, i.e. in the far field for N_a = 10^{17} cm^{-3} and a high surface recombination velocity (5 10^3 $m{\cdot}s^{-1}$); (c, d) in high-injection conditions, i.e. for d=10 nm, N_a = 10^{15} cm^{-3} and a low surface recombination velocity (50 $m{\cdot}s^{-1}$). Simulations made using the FDD model.

2. Detailed analyses about the optimum acceptor doping density

2.1. Existence of an optimum acceptor doping density

In order to give a detailed explanation for the existence of an optimum acceptor doping density, the far-field configuration is selected. Supplementary Figure S4 (a) shows clearly that the depletion region becomes larger as the acceptor doping density decreases. Initially almost fully (99%) in the n-region when N_a = 10^{19} cm^{-3}, the depletion region moves towards the p-region until it fully covers it when N_a = 10^{15} cm^{-3} (and below). When the density of acceptor dopants is reduced, electron mobility increases until reaching a plateau (see Supplementary Fig. S4 (b)). As a result, the photogenerated electrons in the p-region are collected more easily and in turn the

short-circuit current density increases until it saturates (see Supplementary Fig. S4 (c)). But Supplementary Fig. S4 (d) shows the drop of the built-in voltage (V_{bi}) that comes with lowering the doping density. As a result, the dark current raises for smaller applied voltages, in such a way that both the open-circuit voltage (V_{oc}, at which recombination balances generation) and voltage at the maximum power point ($V_{\max}$) drop with the same slope as the built-in voltage. The resulting current density-voltage (J-V) and power density voltage (P-V) are shown on Supplementary Fig. S5 for a set of acceptor doping densities comprised between 10^{14} cm^{-3} and 10^{19} cm^{-3}. There is clearly an observable optimum acceptor doping density (10^{18} cm^{-3}) at which the electrical power at the maximum power point is the largest.

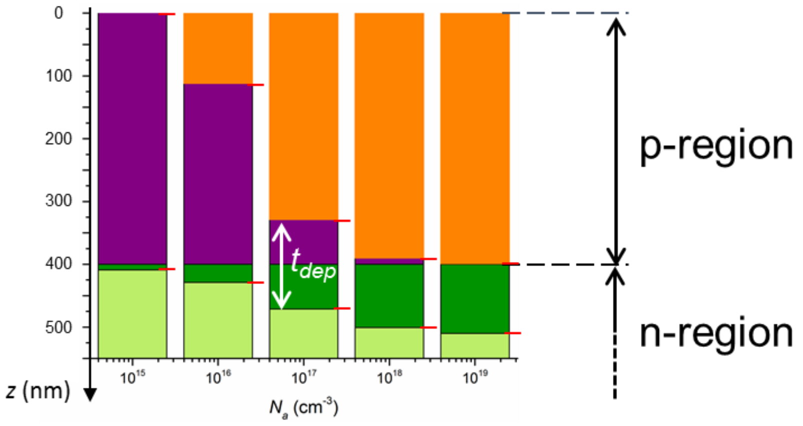

(a)

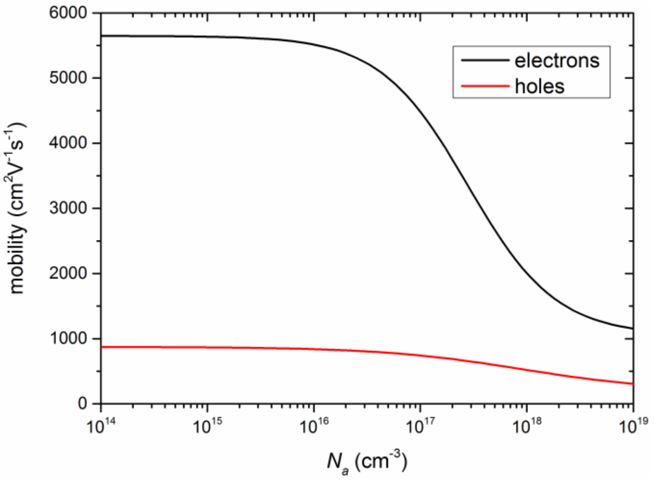

(b)

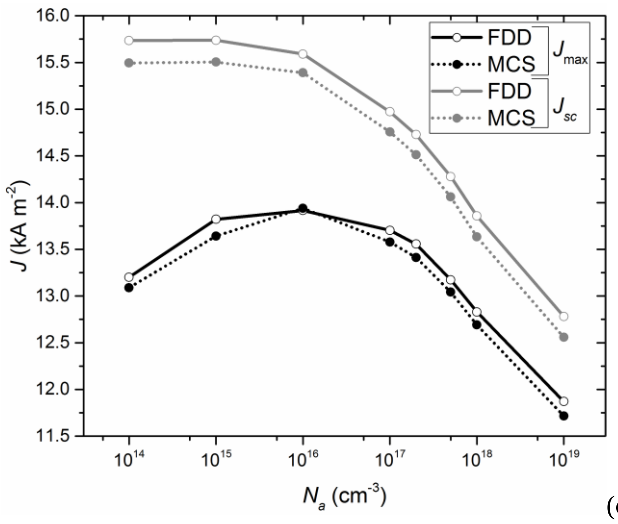

(c)

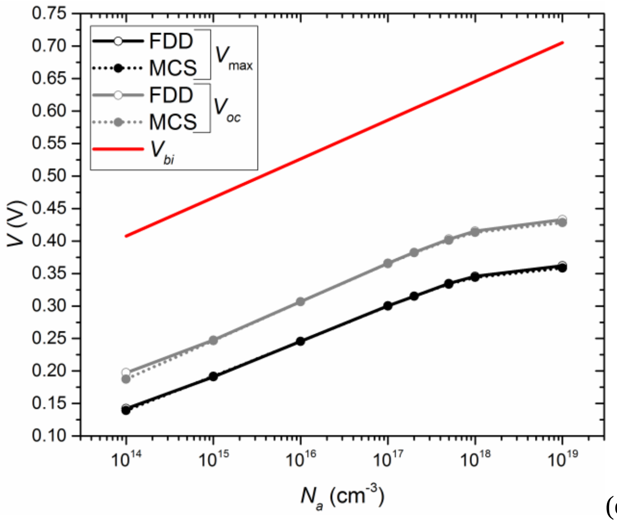

(d)

Supplementary Figure S4. Variations with the acceptor doping density (N_a) of the: (a) location in the cell of the depletion region at short circuit (V = 0), providing its thickness; (b) minority carrier electron mobility; (c) current density at short circuit (J_{sc}) and at the maximum power point ($J_{\max}$); (d) voltage at open circuit (V_{oc}) and at the maximum power point ($V_{\max}$). Simulations made using MCS and the FDD models, except for (a) where only the MCS model has been used. (a), (c), (d): calculations in the far-field configuration for N_d = 10^{17} cm^{-3} and $S_{n,p}$ = 5 10^3 $m \cdot s^{-1}$.

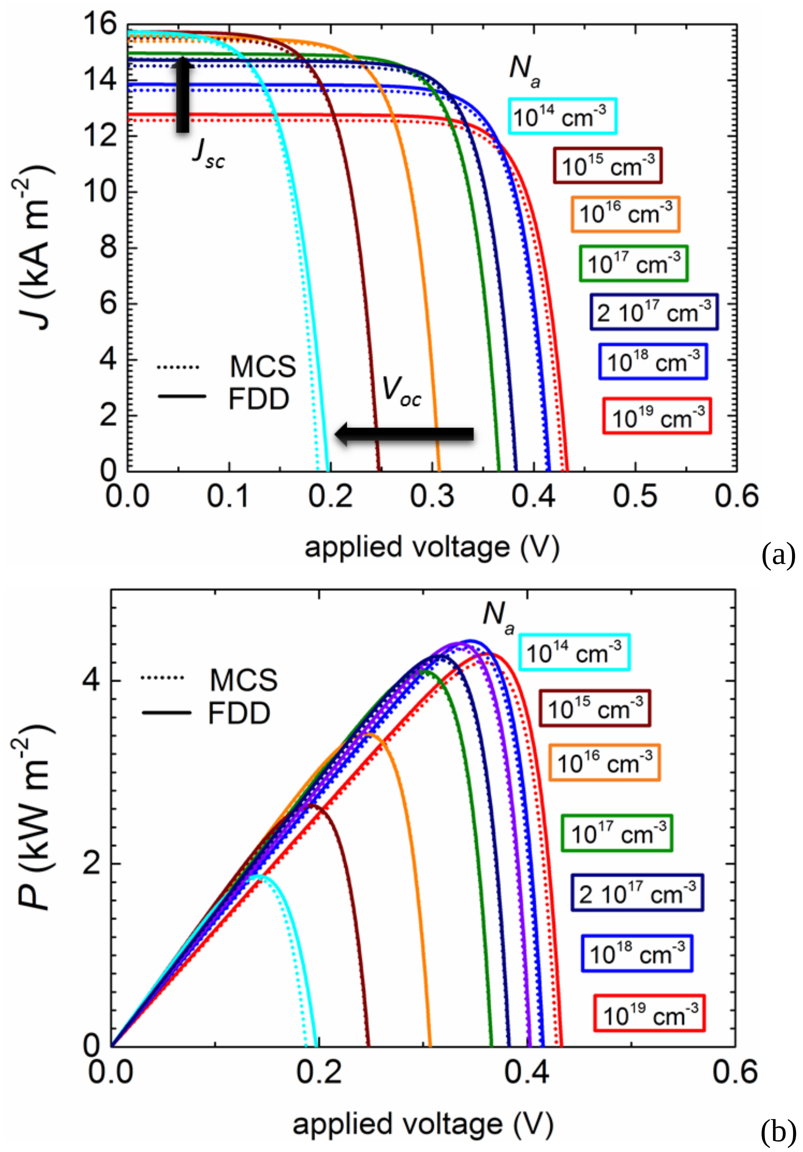

Supplementary Figure S5. (a) J-V and (b) P-V characteristics for a set of acceptor doping densities comprised between 10^{14} cm^{-3} and 10^{19} cm^{-3}, for a far-field radiator-to-cell distance, $N_d = 10^{17}$ cm^{-3} and $S_{n,p} = 5\ 10^3$ m·s^{-1}.

2.2. Change in optimum acceptor doping density as a function of the radiator-to-cell distance

In order to give a detailed explanation for the change in optimum acceptor doping density with radiator-to-cell distance, the case of a high surface recombination velocity ($S_{n,p} = 5\ 10^3$ m·s^{-1}) is selected. Variations of the EHP generation rate with depth in the cell is the main driver of the change in optimum density. Where and how many EHP are generated has an impact on how

many of them are collected (the current density) at the voltage of the maximum power point. However, it is convenient to analyze the variations of the short-circuit current density (J_{sc}), open-circuit voltage (V_{oc}) and fill factor (*FF*) - the maximum electrical power $P_{\max}$ being equal to J_{sc} V_{oc} *FF* - as a function of the acceptor doping density and for the three radiator-to-cell distances. As mentioned above, the short-circuit current density increases when the acceptor doping density is lowered, until reaching a saturation. This is because the depletion region becomes thicker and closer to the front surface of the cell, and mobility of electrons gets larger. But Supplementary Fig. S6 (a) shows that the slope of that short-circuit current rise depends on the radiator-to-cell distance. The largest slope is found when d=10 nm. This is consistent with the spatial distribution of the EHP generation rate (Fig. 2): when the depletion region is closer to the front surface, an increased amount of generated EHP is available to contribute to the photocurrent. Then it is interesting to notice that even though the short-circuit current density is of course larger at d=100 nm than in the far field, the slope of short-circuit density with acceptor doping density is larger in the far-field configuration. This is consistent with the EHP generation rate profile (Fig. 2) which shows that EHP generation grows at a larger rate in the far field than at d=100 nm when approaching the front surface of the cell. These observations are specific to the Drude radiator, since penetration depths are different for the fully propagative, the frustrated and the surface modes (see e.g. [R2]). As a consequence, the far-to-near-field changes in radiation regime lead to differences in the EHP generation rate spatial profile, which in turn rule the photocurrent.

Since the drop in open-circuit voltage and fill factor is almost the same for all three radiator-to-cell distances and N_a comprised between 10^{17} cm^{-3} and 10^{19} cm^{-3} (Supplementary Fig. S6 (b) and (c)), when decreasing the acceptor doping density, the larger the slope in short-circuit current density, the smaller the optimum acceptor doping density ($N_{a,opt}$). Hence $N_{a,opt}$ (d=10 nm) = 2 10^{17} cm^{-3} < $N_{a,opt}$(far field) = 10^{18} cm^{-3} < $N_{a,opt}$(d=100 nm) = 2 10^{18} cm^{-3}. This hierarchy is found for a high surface recombination velocity: for lower values, additional phenomena are involved.

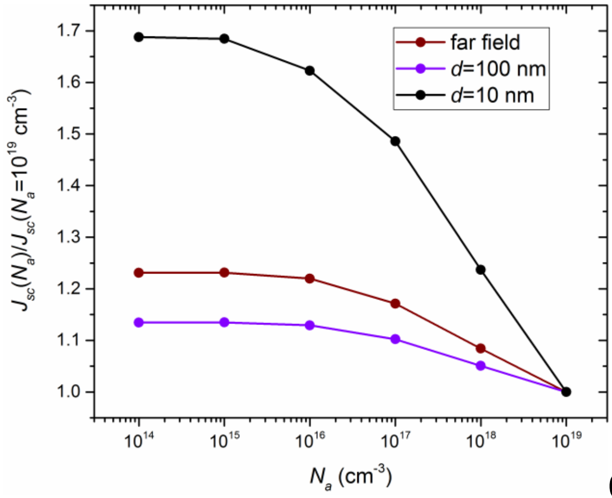

(a)

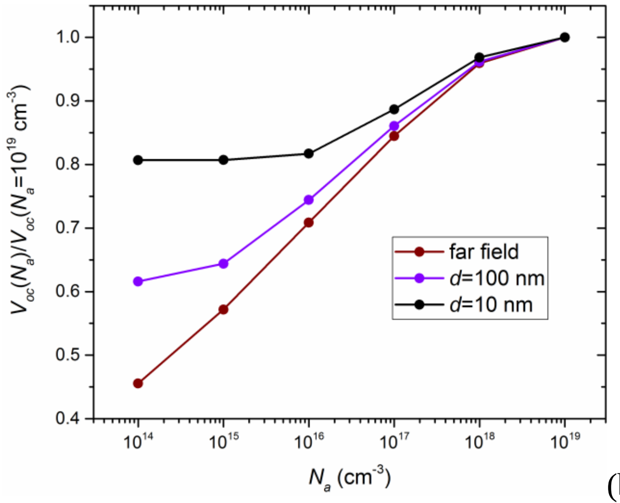

(b)

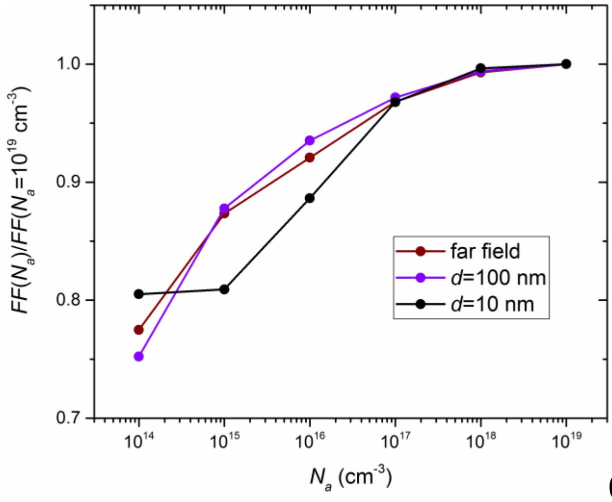

(c)

Supplementary Figure S6. Variations with the acceptor doping density (N_a) of the: (a) short-circuit current density; (b) open-circuit voltage; (c) fill factor; all normalized at N_a = 10^{19} cm^{-3} for three radiator-to-cell distances and $S_{n,p}$ = 5 10^3 m·s^{-1}. For N_a = 10^{19} cm^{-3}, J_{sc}(f-f) = 12.78 kA·m^{-2}, J_{sc}(d=100 nm) = 159.24 kA·m^{-2} and J_{sc}(d=10 nm) = 2113.34 kA·m^{-2}. V_{oc}(far field) = 0.43 V, V_{oc}(d=100 nm) = 0.49 V and V_{oc}(d=10 nm) = 0.57 V. FF(far field) = 0.776, FF(d=100 nm) = 0.798 and FF(d=10 nm) =0.81.

References

R1. Sze, S.M. & Ng, K.K. Physics of semiconductor devices. Third edition, John Wiley & Sons, Hoboken (2007).

R2. Blandre, E. *et al*. Spatial and spectral distributions of thermal radiation emitted by a semi-infinite body and absorbed by a flat film. AIP Advances **5**, 057106 (2015).
